# Special Issue “Alcohol and Inflammation”

**DOI:** 10.3390/ijms27146474

**Published:** 2026-07-21

**Authors:** Candelaria Martín-González, Emilio González-Reimers

**Affiliations:** 1 Departamento de Medicina Interna, Facultad de Medicina, Universidad de La Laguna, 38200 San Cristóbal de La Laguna, Spain; egonrey@ull.edu.es; 2 Servicio de Medicina Interna, Complejo Hospitalario Universitario de Canarias, 38320 San Cristóbal de La Laguna, Spain

The role of inflammation in several manifestations of alcoholism has been a matter of debate for many years [1]. In chronic excessive drinkers, the induction of the microsomal ethanol oxidizing system (MEOS) linked to the activity of cytochrome P450 2E1 (CYP 2E1) leads to the increased production of reactive oxygen species (ROS), which, in addition to other effects, may activate the transcription factor NF-κB [2] and provoke an increased secretion of proinflammatory cytokines. Moreover, ethanol consumption increases gut permeability, so that organisms of the intestinal microbiota, especially Gram-negative bacteria, may enter the portal tract and reach the liver Kupffer cells and macrophages [3,4]. After binding on toll-like receptors (TLR), especially TLR4 [5], they further activate NF-κB transcription factors (among other pathways), increasing the secretion of inflammatory cytokines, especially TNF-α, IL-8, IL-6, and IL-1β. These cytokines are involved in the pathogenesis of many systemic alterations observed in excessive drinkers. Proinflammatory cytokines (especially IL-8) and chemokines (particularly, CXCL1), also induced by the activated macrophages and Kupffer cells, attract neutrophils [6], whereas IL-1β is a potent activator of liver fibrosis, mediated by its effect on hepatic stellate cells [7]. In addition, a proportion of gut-derived Gram-negative bacteria escape the liver filter and enter the systemic circulation, widely expanding the inflammatory response and contributing to the multisystemic manifestations of alcoholism.

Within this framework, several contributions to this Special Issue emphasize different aspects of the pathogenesis of alcohol-induced inflammation. As pointed out by Dukic et al. [8], ethanol consumption is associated with increased intestinal permeability that allows the entering of molecules derived from intestinal microbiota into the portal circulation, triggering an initial response by liver macrophages and Kupffer cells. Based on the importance of microbiota in the pathogenesis of alcoholic liver disease, the authors comment on diverse interventions on intestinal flora, in line with therapeutic approaches carried out by other authors [9,10].

Partly in relation to increased gut permeability, alcoholic patients are prone to develop infections, which are usually more severe than those observed in non-alcoholics, especially when alcoholic liver disease ensues [11]. Pulmonary infections are especially common and severe [12], mostly due to altered systemic and local immune response, affecting both specific immune cells and myeloid cells [13]. However, the precise mechanisms involved are not fully known. Among those pathways potentially affected by ethanol, Hu et al. [14] have studied the importance of alteration in the adenyl cyclase-cAMP system in myeloid cells. Adenyl cyclase (AC), an enzyme with 10 isoforms among mammals, increases the production of cAMP. cAMP is a key inhibitory mediator of both innate and specific immune responses, impairing phagocytosis, microbicide activity, and inflammatory response [15]. Adenyl cyclase 7 (AC7) is especially sensitive to the action of ethanol, as ethanol is a potent stimulator of its activity [16], therefore theoretically depressing immune defensive mechanisms. Whole-body AC7 knockout (KO) mice have a high mortality; so, an experimental strain was designed in which AC7 KO only affected myeloid cells. Animals with this genotype were then treated with ethanol or water and were exposed or not to a lipopolysaccharide (LPS) challenge. As expected, LPS challenge reduced the survival of alcoholic-treated wild-type (WT) animals, but not the group of AC7KO animals. The mortality of the AC7KO animals was higher than that of WT animals, independently of whether they were treated or not with ethanol, underscoring the defensive role of AC7. However, surprisingly, ethanol treatment was associated with a better survival after LPS challenge among AC7KO mice than among wild-type animals, as if ethanol could exert a certain degree of protection against mortality induced by the lack of AC7.

As expected, LPS intraperitoneal injections caused a rise in proinflammatory cytokines in the lungs and liver. In general, cytokine expression after acute ethanol administration was far higher among AC7KO male animals than among WT male animals. The effect of AC7 on cytokine expression seems to be organ-specific, and some differences were also observed in this experimental model among male and female animals. Female hormones seem to protect against inflammation, a finding in accordance with some observations [17], although opposite results have been also reported [18,19]. In summary, as stated by the authors, the inflammatory effect of ethanol—at least on cytokine expression—strongly differs in relation to the pattern of alcohol exposure (acute or chronic), and also to the tissue examined. This study illustrates the complexity of the mechanisms involved in ethanol-mediated alteration in inflammatory and immune response, a field that requires further research.

Three pathways are involved in ethanol metabolism: alcohol dehydrogenase (ADH), MEOS, and catalase, transforming ethanol into acetaldehyde, an intermediate product. Acetaldehyde is a toxic compound that should be rapidly transformed into acetate, as the final product of ethanol metabolism.

Chronic ethanol consumption in high doses leads to the induction of the MEOS system, but the quantitatively most important enzymatic pathway is that mediated by ADH. There are many isoforms of ADH, with varied properties [20]. Indeed, ADH consists of two subunits, which can be combined as homodimers or heterodimers, leading to a variety of enzymes with different kinetics and metabolic activity. Based on these criteria, several ADH classes have been identified, with marked differences in their velocity to transform ethanol into acetaldehyde [21]. In addition, there are also several isoforms of aldehyde dehydrogenase, which convert acetaldehyde into acetate. Therefore, there is an important variability among individuals with respect to the amount of acetaldehyde generated in a given time after ethanol consumption, as well as in the period required to convert the toxic acetaldehyde into harmless acetate. Acetaldehyde is mainly responsible for the deleterious effects of ethanol drinking, including the increased production of proinflammatory cytokines, directly involved in liver damage and carcinogenesis [22], since acetaldehyde may form adducts with DNA molecules. Therefore, the combination of an ADH isoform providing a large amount of acetaldehyde together with a low-activity ALDH variant may be especially dangerous. In this issue, Tadokoro et al. [23] reports a high prevalence of the ADH1B*2 polymorphism among people of Eastern Asia, combined with heterozygous carriers of the slow-reacting ALDH2 polymorphism. The high acetaldehyde concentrations derived from such a combination may provoke flushing and other unpleasant side effects; so, affected individuals tend to drink less, and therefore, this polymorphism is associated with a reduced risk of liver cirrhosis. This genotype combination is highly prevalent among the East Asian population [24], and it is associated with a high risk for malignancies of the upper digestive tract (larynx, pharynx, esophagus, and oral cavity). Based on the different combinations of ADH and ALDH polymorphisms, Tadokoro et al. provide a classification useful for identifying individuals at different risk levels, opening the possibility for personalized prevention through genetic testing.

The metabolism of ethanol via the alcohol/aldehyde dehydrogenase pathway consumes NAD and generates NADH, leading to a depletion of the former [25]. This altered NAD/NADH ratio has metabolic consequences: biochemical processes in which NAD is needed become depressed, while those in which NADH is needed become enhanced. This is the case for metabolic pathways involved in lipid metabolism. An excess of the NADH/NAD ratio enhances lipogenesis but impedes lipolysis [26]. This is one of the mechanisms by which ethanol causes hepatocyte fat accumulation in the early stages of alcoholic liver disease. However, there are additional pathogenetic pathways directly related to inflammation, partly derived from TLR-4 activation by intestinal bacteria. TLR-4 activation induces TNF-α secretion by sensitized Kupffer cells and, among other functions, upregulates SREBP-1c (Sterol Regulatory Element-Binding Protein-1c) activity [27,28]. Macrophages and Kupffer cells express GPR-120 (FFA4), a receptor for free fatty acids, including ω-3 polyunsaturated fatty acids (PUFA), whose activation exerts potent anti-inflammatory actions, including decreased expression of SREBP-1c, a molecule that, in addition to its lipogenic effect, induces cytokine secretion and liver inflammation [29].

It is well known that ω-3 PUFAs may block the activation of Kupffer cells, interfering with the development of non-alcoholic steatohepatitis and the progression to more advanced forms of liver injury. Although this statement is not undisputed [30,31,32], this matter has been extensively studied in non-alcoholic fatty liver disease [33]. In this Special Issue, Kang et al. [34] showed that the administration of PUFA reduces alcoholic steatohepatitis, AST/ALT levels, and serum triglyceride levels. These actions were not observed when a selective FFA4 antagonist was administered. As expected, PUFA reduced SREBP1c gene expression via FFA4 activation (the reduction was not observed in FFA4 knockout mice) and also reduced the activity of fatty acid synthase and glycerol phosphate acyl transferase, with the amelioration of steatosis. These changes were not observed in FFA4 knockout mice. In addition, PUFA activation of FFA4 provoked a decrease in TNF-α, cyclooxygenase 2 (COX-2), and NLR family pyrin domain containing 3 (NLRP-3), a receptor able to detect PAMPs or DAMPs (pathogen- and damage-associated molecular patterns), involved in cytokine secretion and pyroptosis. Kang’s results shed light on the debated effects of PUFA on alcoholic liver disease [35,36], showing a benefit after short-term (2-week) alcohol administration. This conclusion is in accordance with the generalized view of increased SREBP activity in alcoholic fatty liver, accompanied by a decrease in the lipolytic PPAR-α, a pattern also observed in obese individuals with PUFA depletion and insulin resistance. Additionally, alcoholics may show a deficient intake of PUFA [37], and a low dietary PUFA intake increases the severity of alcoholic liver disease [38].

Chronic ethanol consumption leads to liver damage, which may have two important consequences related to the systemic effects of ethanol. Firstly, portocaval shunting develops, facilitating the escape of intestinal bacteria from the hepatic filter into the systemic circulation; on the other hand, liver function derangement impairs, among many other metabolic pathways, the liver’s capacity to transform ammonia into urea, and hyperammonemia ensues. However, liver function impairment is not a necessary condition for hyperammonemia, since acetaldehyde may directly interfere with metabolic shuttles involved in ammonia metabolism at the mitochondrial level [39], and excessive drinkers are exposed to increased amounts of endotoxin even with preserved liver function. Indeed, endotoxemia and hyperammonemia, among other factors, contribute to the sarcopenia observed in alcohol misusers.

It has been shown that endotoxemia may inhibit the mTORC1 pathway and promote autophagy [40]. mTORC1 is a pro-anabolic signaling complex that promotes cell growth by responding to growth factors, such as insulin or IGF-1, and amino acids. The Lamtor/Ragulator is a regulatory complex that participates in the sensing of amino acids and the activation of mTORC1 [41,42]. Activation of mTORC1 in the presence of these factors (insulin, amino acids) inhibits autophagy [43]; however, as noted, endotoxemia inhibits the muscle-anabolic effects of mTORC1. In addition, hyperammonemia induces myostatin synthesis [44]. Myostatin potentiates muscle fiber apoptosis and inhibits protein synthesis.

Epidermal growth factor (EGF) is liberated by Brunner’s glands and exerts important systemic functions as a factor involved in cell growth and proliferation. Specifically, EGF protects the intestinal barrier: it acts on tight junctions, reduces intestinal permeability, and favors proliferation of intestinal stem cells located at the intestinal crypts [45], globally assuring the preservation of the intestinal barrier and preventing bacterial translocation [46], although some discordance exists regarding this last finding [47]. Based on these two main facts—i.e., the relation of sarcopenia with endotoxin and the protective effects of EGF on intestinal permeability—Xiao et al. [48] showed that EGF reduced myostatin and the expression of several proteins involved in muscle catabolism, but it did not revert the reduced grip strength and reduced cross-sectional areas of muscle fibers caused by ethanol. The effects on myostatin and proteins involved in muscle catabolism were accompanied by a reduction in ethanol-induced damage to the intestinal barrier and also improved liver histology, liver enzyme levels, cytokine levels, and a reduction in the activity of liver inflammatory pathways (MyD88). Changes in the serum amino acid profile and microbiota composition were also observed in EGF-treated animals. Undoubtedly, this study holds promise regarding the usefulness of EGF supplementation in alcoholics, not only for muscle damage but also as a protective factor against liver damage and endotoxemia (Figure 1).

In a clinical setting, the terms sarcopenia, sarcopenic obesity, and osteosarcopenic obesity (OSO) were coined to define a situation in elderly people strongly associated with frailty and increased mortality [49]. These frequently associated features among the elderly are also common in alcoholics; so, Martín-González et al. [50] analyzed the prevalence of OSO among excessive drinkers and its relationship with proinflammatory cytokines (TNF-α, IL-6, IL-8) and vitamin D. A high prevalence of OSO was found among the 115 patients included, especially regarding OSO obesity (60%), OSO osteopenia (55.65%), and OSO lean mass (60.17%). OSO was far more frequent among excessive drinkers than among an age-matched control group. Excessive drinkers showed higher values of IL-6 and IL-8, which were closely related to reduced muscle strength. These results underscore the importance of inflammation in ethanol-mediated muscle damage. Interestingly, vitamin D deficiency showed a close correlation with OSO handgrip strength, suggesting its pathogenetic involvement in sarcopenia in patients with alcohol use disorder, as other studies have also suggested [51].

Ethanol-mediated inflammation may also involve the brain, as shown by several studies [52]. Indeed, prenatal ethanol exposure and its accompanying inflammation seem to play a major role in a variety of neurological disorders [53], with a clinical expression during growth or even, possibly, in adulthood [54]. Chemokines, in general, can be considered mediators of inflammation, since their expression, including that of CXCL-16, increases in inflammatory conditions [55]. In this Special Issue, Padilla-Valdez et al. [56] centered their study on the hippocampus, a brain region heavily involved in memory, learning, and cognition. They show that prenatal alcohol exposure leads to changes in the expression of the chemokine CXCL-16 along different areas of the hippocampus (dentate gyrus, CA1 and CA3), with strikingly different behavior between male and female offspring. Changes in CXCL-16 expression also vary during the postnatal period. This contribution may be important for future studies, given the protean effects of this chemokine, possibly involved in neuroplasticity and neuroprotection [57,58], and underscores the importance of alcohol-mediated inflammation in hippocampal development.

Synaptic plasticity, the ability to modulate and develop synaptic connections in response to stimuli, is an essential process in learning, memory, and cognition [59]. However, specific neuronal circuits are involved in the development of alcohol dependence and promote the “learning” of alcohol-seeking habits, as is the case, for instance, of long-term potentiation in the dorsomedial striatum [60], although changes in plasticity—strengthening or weakening of synapses—vary by brain region and exposure duration (acute vs. chronic) [61]. Several pathways associated with neurotransmitter activity are involved and logically modulated by the expression of different genes. Legaki et al. [62], in this Special Issue, analyzed changes in the expression of genes related to synaptic plasticity associated with alcohol abuse, in order to identify differences between patients and controls, to identify a genetic profile associated with relapse after an alcohol-detoxification intervention program, and to assess differences in the gene expression induced by the intervention program in adult (18–70 years) individuals affected by alcohol overuse and non-drinker controls. Interestingly, several of the genes analyzed also play essential roles in inflammatory pathways. This is the case of MMP-9, a zinc-dependent enzyme that plays a critical role in inflammation and wound healing, clearly upregulated in the study by Legaki et al. [62]. Chronic MMP-9 overexpression causes pathological inflammation, tissue destruction, and fibrosis. Several genes of the EGR family are also important regulators of inflammation and immune response, but they are also heavily involved in synaptic plasticity, being upregulated in the patients included in Legaki’s study, as well as CREB1, an anti-inflammatory NF-κB inhibitor and promoter of IL-10 synthesis [63]. All these findings strongly support the existence of a link between alcohol dependence and alcohol-seeking habits, on the one hand, and systemic inflammation, on the other; a new and complex field of knowledge related to alcoholism (Figure 2).

Therefore, in this Special Issue, a few examples of the protean multisystemic effects related to excessive ethanol consumption are reviewed. The alteration of intestinal permeability, leading to an increase in circulating LPS, able to trigger activation of inflammatory pathways in many organs, together with the increased ROS generation from the metabolism of ethanol itself, are key features involved in the systemic effects of excessive drinking. As illustrated by the several contributions reported in this Special Issue, many open questions still remain, which deserve further research.

## Figures and Tables

**Figure 1:**
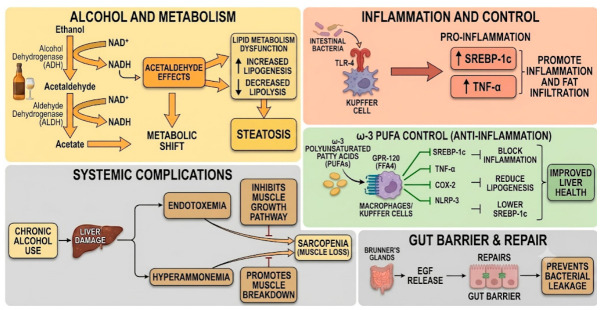
Mechanisms of ALD, inflammation, and systemic complications. Ethanol metabolism alters the NADH/NAD ratio to induce steatosis, while gut-derived TLR-4 activation triggers Kupffer cell inflammation. Conversely, ω-3 PUFAs activate the FFA4 receptor to block SREBP-1c and pro-inflammatory cytokines, reducing liver injury. Finally, chronic damage leads to endotoxemia and hyperammonemia, which collectively inhibit muscle growth and promote sarcopenia.

**Figure 2:**
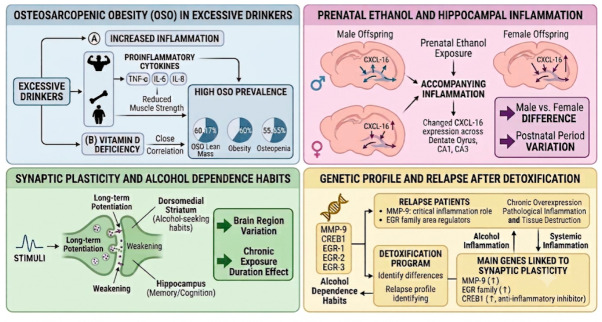
Systemic effects of alcohol on musculoskeletal integrity, neuroinflammation, and synaptic plasticity. Excessive drinking triggers pro-inflammatory cytokines (TNF-α, IL-6, IL-8) and vitamin D deficiency, driving a high prevalence of osteosarcopenic obesity (OSO) and muscle weakness. Concurrently, prenatal and chronic alcohol exposure induce regional neuroinflammation—mediated by altered hippocampal CXCL-16 expression with distinct sex-based and postnatal profiles—while chronic upregulation of synaptic plasticity genes (e.g., MMP-9, EGR family, CREB1) links alcohol-seeking habits to pathological systemic inflammation.
